# Supplementary material for: Complex‐centric proteome profiling by SEC‐SWATH‐MS
Source: Mol Syst Biol. 2019 Jan 14;15(1):e8438. doi: 10.15252/msb.20188438 (PMC6346213; doi:10.15252/msb.20188438)
Supplement: Supplementary file 7 — Dataset EV6 [file MSB-15-e8438-s007.zip › feature_plots_bioplex/Q7Z5B4.pdf]

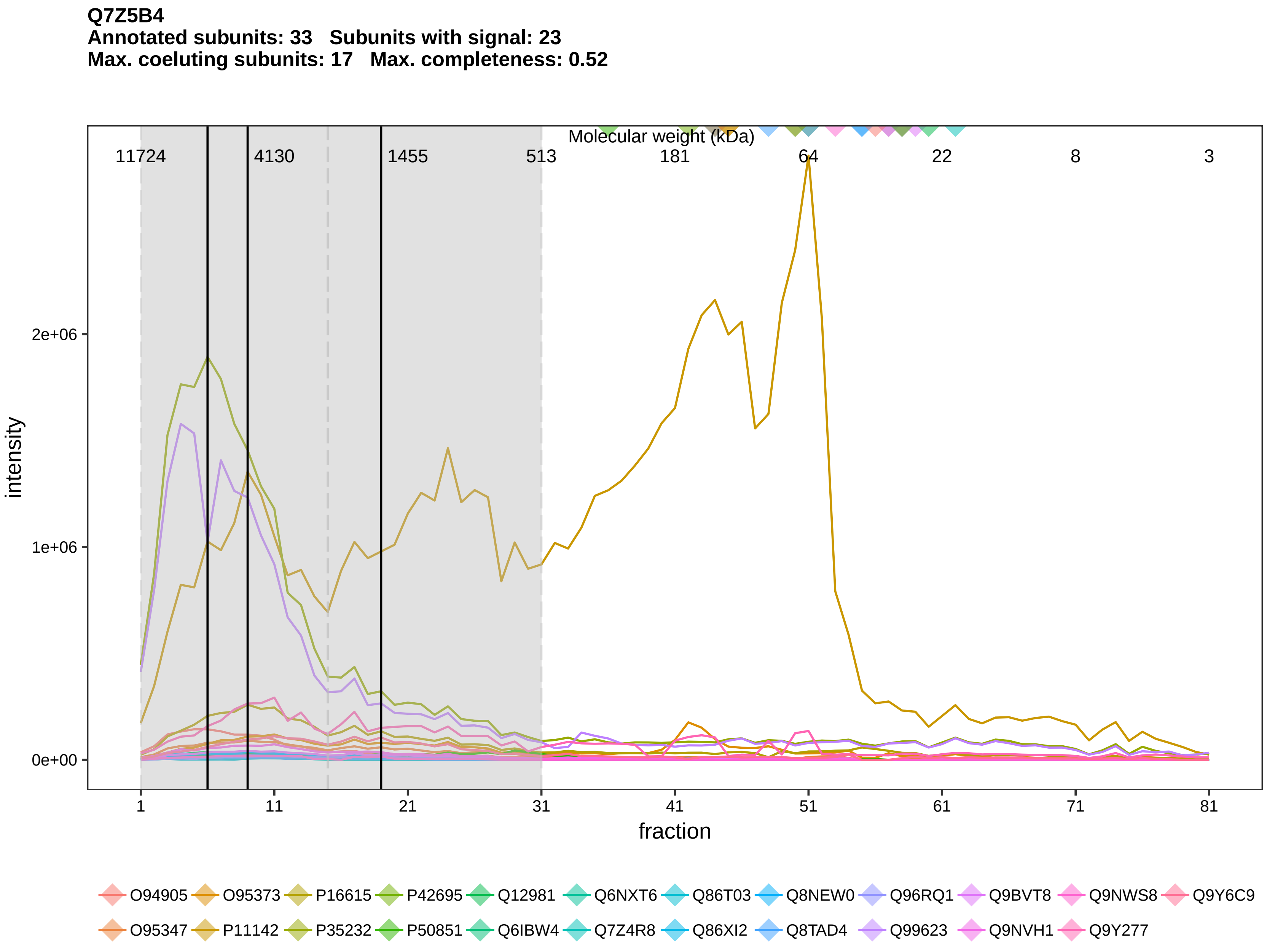
Q7Z5B4
Annotated subunits: 33 Subunits with signal: 23
Max. coeluting subunits: 17 Max. completeness: 0.52
Molecular weight (kDa)
11724
4130
1455
513
181
64
22
8
3
intensity
2e+06
1e+06
0e+00
1
11
21
31
41
51
61
71
81
fraction
O94905
O95373
P16615
P42695
Q12981
Q6NXT6
Q86T03
Q8NEW0
Q96RQ1
Q9BVT8
Q9NWS8
Q9Y6C9
O95347
P11142
P35232
P50851
Q6IBW4
Q7Z4R8
Q86XI2
Q8TAD4
Q99623
Q9NVH1
Q9Y277
